# Factors associated with childbirth self-efficacy in Australian childbearing women

**DOI:** 10.1186/s12884-015-0465-8

**Published:** 2015-02-13

**Authors:** Lianne Schwartz, Jocelyn Toohill, Debra K Creedy, Kathleen Baird, Jenny Gamble, Jennifer Fenwick

**Affiliations:** 1 School of Nursing and Midwifery, Griffith University, University Drive, Meadowbrook, QLD 4131 Australia; 2 Griffith Health Institute, Griffith University, University Drive, Meadowbrook, QLD 4131 Australia; 3 Gold Coast University Hospital, Parklands Drive, Southport, QLD 4215 Australia

**Keywords:** Pregnancy, Childbirth self-efficacy, Parity, Caesarean section, Fear, Depression

## Abstract

**Background:**

Childbirth confidence is an important marker of women’s coping abilities during labour and birth. This study investigated socio-demographic, obstetric and psychological factors affecting self-efficacy in childbearing women.

**Method:**

This paper presents a secondary analysis of data collected as part of the BELIEF study (Birth Emotions – Looking to Improve Expectant Fear). Women (n = 1410) were recruited during pregnancy (≤24 weeks gestation). The survey included socio-demographic details (such as age and partner support); obstetric details including parity, birth preference, and pain; and standardised psychological measures: CBSEI (Childbirth Self-efficacy Inventory), W-DEQ A (childbirth fear) and EPDS (depressive symptoms). Variables were tested against CBSEI first stage of labour sub-scales (outcome expectancy and self-efficacy expectancy) according to parity.

**Results:**

CBSEI total mean score was 443 (SD = 112.2). CBSEI, W-DEQ, EPDS scores were highly correlated. Regardless of parity, women who reported low childbirth knowledge, who preferred a caesarean section, and had high W-DEQ and EPDS scores reported lower self-efficacy. There were no differences for nulliparous or multiparous women on outcome expectancy, but multiparous women had higher self-efficacy scores (p < .001). Multiparous women whose partner was unsupportive were more likely to report low self-efficacy expectancy (p < .05). Experiencing moderate pain in pregnancy was significantly associated with low self-efficacy expectancy in both parity groups, as well as low outcome expectancy in nulliparous women only. Fear correlated strongly with low childbirth self-efficacy.

**Conclusion:**

Few studies have investigated childbirth self-efficacy according to parity. Although multiparous women reported higher birth confidence significant obstetric and psychological differences were found. Addressing women’s physical and emotional wellbeing and perceptions of the upcoming birth may highlight their level of self-efficacy for birth.

**Trial registration:**

Australian New Zealand Controlled Trials Registry ACTRN12612000526875, 17^th^ May 2012.

## Background

Confidence in labour and birth, also known as childbirth self-efficacy, has been identified as an important marker of women’s coping abilities during labour [1]. According to Bandura [2], self-efficacy reflects personal beliefs about behaviour that influence outcomes. Self-efficacy is influenced by individuals’ past experiences in mastering the situation at hand, the vicarious experiences of others, verbal persuasion, and degree of emotional and physiological arousal. There are two primary aspects to self-efficacy. Outcome expectancy refers to trusting that a behaviour will lead to a certain outcome. Self-efficacy, on the other hand, is the individual’s belief that they are able to perform that behaviour successfully in a particular context [3]. The difference is crucial to understand as people may believe a certain behaviour to be effective, but not have faith in their ability to perform it [4].

Research on childbirth self-efficacy has focused on primarily homogenous samples of well-educated nulliparous women attending childbirth classes [5-10]. Relatively few studies have included multiparous women [3,11,12]. Furthermore, we know relatively little about childbirth self-efficacy in large representative samples of childbearing women. This paper reports on socio-demographic, obstetric and psychological factors associated with childbirth self-efficacy in nulliparous and multiparous childbearing women in Australia.

### Factors associated with childbirth self-efficacy

#### Socio-demographic factors

There is inconclusive evidence around socio-demographic factors and childbirth self-efficacy. No consistent link has been found between self-efficacy and age [13-16], co-habitation or occupational status [15], or educational level [13,15]. High self-efficacy scores have been associated with healthier psychosocial adaptation following childbirth and stronger identification with the role of motherhood [9].

### Obstetric factors

Similarly, evidence linking obstetric factors and childbirth self-efficacy has been inconsistent. In regards to parity, early research has revealed high self-efficacy scores for nulliparous women [11] and multiparous women [3]. High self-efficacy has been associated with a previous positive birth experience [12,17] while low self-efficacy has been reported in women experiencing potentially negative events such as a previous caesarean section [11].

In relation to birth choice, lower self-efficacy scores have been related to a stronger preference for Elective Repeat Caesarean Section (ERCS) [11]. More recent research, however, has found no such relationship between self-efficacy and birth mode choice [15,18]. One study compared a specialised ‘next birth after caesarean’ (NBAC) clinic with standard care and found that the increased knowledge and information about birth options provided in the service increased women’s childbirth self confidence levels, however, this did not translate to a higher number of vaginal births [19].

Pain is often seen as a key variable in the experience of birth, and is certainly one that women appear to focus on in anticipation of the event. The relationship between confidence about the anticipated event of labour and birth and perceptions and experiences of pain during childbirth has been closely examined over the last 30 years. An early study in 1983 by Manning and Wright [20] found evidence of lower pain perceptions and less pain medication use for women with high self-efficacy. The bulk of subsequent research has also related higher self-efficacy scores to lower pain perceptions, or less pain in labour [6,16,21,22]. One exception is a small study focusing only on early labour pain scores that found no correlation between pain and self-efficacy [5]. In addition, no relationship between use of medication for pain relief and level of self-efficacy has been found in later research [6,16,23].

### Psychological factors

Self-efficacy in childbearing women has been linked to fear, anxiety, and post-traumatic stress symptoms. One of the strongest correlations is between low self-efficacy and fear of childbirth [1,8,15]. This relationship seems to persist independent of demographic, psycho-social, or other factors. Prenatal anxiety has also been related to low self-efficacy in nulliparous women [5,9] although Australian researchers Drummond and Rickwood [12] found no significant relationship in a combined nulliparous and multiparous sample.

In another study focusing on prevalence and predictors of women's experience of psychological trauma during childbirth, Soet and colleagues [24] found that low self-efficacy for coping with the first and second stages of labour was associated with the development of PTSD symptoms in women following birth. Some 10 years later the work of Goutaudier et al. [25] contradicted this finding. Using the Child Birth Self-Efficacy Inventory (CBSEI) for retrospective measurement of women’s feelings of self-efficacy during labour and birth, the researchers reported no association between levels of self-efficacy and PTSD. Postnatal women, however, rated their perceived ability to cope with the actual past event differently than their anticipated ability to cope [25].

### Aim

While work has been conducted to validate the self-efficacy measure for different populations and nationalities including Australian women [12], there are no parallel Australian studies investigating factors associated with childbirth self-efficacy. Our aim is to identify socio-demographic, obstetric or psychological variables associated with women’s childbirth self-efficacy, and potentially highlight areas to improve women’s childbirth experiences.

## Method

This paper reports on a secondary analysis of data collected as part of the BELIEF (Birth Emotions – Looking to Improve Expectant Fear) study. The protocol for this study has been published [26].

### Participants

There were 2,311 eligible women invited to the Belief study with 1,410 (61%) agreeing to participate. Included were women ≤ 24 weeks gestation, aged 16 and older, able to read, write and understand the English language, with the capacity to consent. If after recruitment women came to expect a perinatal death (e.g. congenital abnormality incompatible) or stillbirth they were given an opportunity to withdraw from the study. If they opted to continue they were offered counselling support and received copies of the BELIEF study newsletters if requested.

The sample was representative of state and national birthing populations as reported previously [27]. Participants had a mean age of 29 years and just over half were multiparous (57%). The large majority were Australian born (74%), most were in a committed relationship (93%), around half the sample had post Year 12 level of education (50%), and around three quarters (72%) were in some form of paid employment.

### Recruitment & settings

Women were recruited from antenatal clinics of three south-east Queensland metropolitan teaching hospitals between May 2012 and June 2013. Combined, the hospitals provide 8, 500 publicly funded births per year.

All women scheduled to attend an antenatal clinic at a participating site received a study flyer by mail with their antenatal booking appointment and could contact the researcher for inclusion in the study. Furthermore, the antenatal clinics were attended by a recruitment midwife each day. All women who met the study criteria were identified by clinic staff and approached to participate by the research midwife. They were provided with written and verbal information about the study and written consent was obtained. Human research ethics approval was obtained from Griffith University and Queensland Health multi-site hospital Human Research Ethics Committee.

### Measures

Questionnaires were completed at time of recruitment in the antenatal clinic or returned by free post to the University research office. Women who had not returned questionnaires were telephoned at two weeks (or if no answer, the secondary contact number was used), and at four weeks an SMS and email were sent to prompt completion of questionnaires by return mail or be completed over the telephone.

### Antenatal questionnaire

The BELIEF antenatal questionnaire sought personal information, obstetric details and completion of psychometric measures. Categorical variables identified in the literature for possible associations to birth self-efficacy were drawn from the BELIEF data for this secondary analysis. Specifically participants’ age, education parity, previous miscarriage and birth preference were extracted. One question to assess pain was drawn from the EuroQol [28]; one question from Drummond and Rickwood’s [12] Childbirth Knowledge Questionnaire and another question from their Social Support/Persuasions Scale were included to assess knowledge; and partner support (refer Table 1). These single item questions were used as they were easily identifiable from the questionnaires and specific to identifying relationships to self-efficacy for the purpose of this study. Psychometric measures were the Childbirth Self-Efficacy Inventory (CBSEI), Wijma Delivery Expectancy Questionnaire (W-DEQ-A) for fear and Edinburgh Postnatal Depression Scale (EPDS) for depressive symptoms.

**Table 1:** **Questions in study extracted from psychometric measures**

Source	Original question	Analysis & reporting
EuroQuol group (28)	Please indicate which statements best describe your own state of health today.	Analysed as dichotomous variable:
		Response (a) versus (b/c) combined
	Pain/discomfort:	
	a. I have no pain or discomfort	
	b. I have moderate pain or discomfort	
	c. I have extreme pain or discomfort	
Childbirth knowledge questionnaire (12)	In comparison to other women, how detailed is your knowledge of childbirth?	Analysed as dichotomous variable:
	a. Much less detailed	Response (a/b) versus (c/d/e) combined
	b. Less detailed	
	c. Same	
	d. More detailed	
	e. Much more detailed	
Social support/persuasions scale (12)	How supportive of this pregnancy is your partner?	Analysed as dichotomous variable:
		Response (a/b/c) versus (d/e) combined
	a. Not at all supportive	
	b. Not very supportive	
	c. Indifferent	
	d. Fairly supportive	
	e. Very supportive	

*The Childbirth Self*-*Efficacy Inventory (CBSEI)* is a 62-item scale requiring responses on a 10-point Likert scale [3]. The CBSEI has four components to capture the specific beliefs and behaviours during the first and second stage of labour. For each stage of labour, items address a woman’s ‘belief’ that a specific behaviour would lead to a given outcome (expectancy outcome), and her conviction that she could indeed ‘perform’ that behaviour (self-efficacy). The four sub-scales indicate levels of high or low childbirth confidence. A higher score indicates a higher level of self-efficacy or outcome expectancy for birth [3]. The CBSEI has been validated for use in the Australian birthing population and reported reliability coefficients for all four subscales are above 0.90 [12].

*Wijma Delivery Expectancy/Experience Questionnaire (W-DEQ)* is a 33-item scale [29]. Respondents rate their expectations before birth (version A) and experiences after birth (version B). Questions are presented in positive and negative formats on a six point Likert scale from 0–5 requiring reverse scoring of positively formulated questions. A score equal to or lower than 37 is considered low fear, a score between 38 and 65 equates to moderate fear and a score equal to or higher than 66 represents a high level of fear [30]. The original authors reported a Cronbach’s alpha of 0.93 in a population of nulliparous and multiparous women [29]. The W-DEQ has been validated in studies with Australian childbearing women [27,31].

*Edinburgh Postnatal Depression Scale (EPDS)* has 10-items and is widely used to screen for probable antenatal and postnatal depression [32,33]. Women select one of four possible responses to each question. Each item is scored from 0 – 3 and summed to produce a total score. The range of scores is from 0 – 30 with higher scores indicating more negative feelings. EPDS scores of >12 in the antenatal or postnatal period have been recommended as an indicator of probable depression but not diagnostic of depression [34].

### Approach to analysis

Descriptive statistics were conducted on all variables. The reliability of each scale used in the study was calculated. Any missing data was not replaced. Cronbach’s alpha values for CBSEI first stage subscales were 0.93 for expectancy outcome and 0.96 for self-efficacy, 0.94 for the W-DEQ and 0.86 for the EPDS. Spearman’s rho was used to determine any correlation between CBSEI, W-DEQ and EPDS scales. Mann Whitney U determined differences between parity groups against CBSEI subscales. Independent samples t-test was used to explore relationships between childbirth efficacy and independent categorical variables (perceived partner support, parity, history of pregnancy loss, equal or less than Year 12 versus more than Year 12 education, perceived birth knowledge compared to peers, moderate or severe pain in second trimester pregnancy, and birth mode preference). Pearson’s r was used to determine relationships between childbirth efficacy and the independent continuous variables of age, W-DEQ and EPDS scores. Correlations between all variables were also analysed with Pearson’s r. Analyses were conducted by parity groups. A significance level of < .05 was used for all analyses.

## Results

A total of 1306 (92.6%) women completed the CBSEI (total mean score = 443, SD = 112.2). However there were additional women who completed specific subscales of the CBSEI with responses being higher for first stage outcome expectancy (n = 1376, 97.6%) and lower for second stage self-efficacy (n = 1335, 94.7%) (refer Table 2). There was a significant difference in total CBSEI self-efficacy scores when analysed by parity, but no difference in total outcome expectancy between nulliparous and multiparous women. Women who had given birth previously recorded higher self-efficacy scores (Table 2).

**Table 2:** **Description of CBSEI and differences between sub-scales by parity**

CBSEI sub-scales		Sub-scales by parity			
CBSEI subscale cronbach alpha	Mean, SD	Parity	Nullip n = 609 (%)	Median	P-value
			Multip n = 801 (%)		
1^st^ Stage (OE)	113.3, 25.6	Nulliparous	602 (99)	118	0.60
.93		Multiparous	774 (97)	118	
1^st^ Stage (SE)	103.3, 30.5	Nulliparous	593 (97)	103	<.001
.96		Multiparous	758 (95)	111	
2^nd^ Stage (OE)	116.7, 32.2	Nulliparous	594 (98)	121	0.15
.96		Multiparous	765 (96)	124	
2^nd^ Stage (SE)	108.9, 35.2	Nulliparous	585 (96)	111	<.001
.97		Multiparous	750 (94)	116	

OE = Outcome Expectancy; SE = Self Efficacy.

There was a strong inter-correlation (rho .64 or higher, p < .001) between the four CBSEI subscales with all Cronbach alpha coefficients >0.90 (see Table 2). This result supported using the subscales of outcome expectancy and self-efficacy for first stage of labour only against all independent variables. The mean score for first stage of labour outcome expectancy subscale for nulliparous (m = 113.6, SD = 26) and multiparous women were similar (m = 113.1, SD = 25.4) with mean scores for the first stage of labour self-efficacy subscale lower in nulliparous women (m = 98.8, SD = 31.7) compared to multiparous women (m = 106.9, SD = 29) indicating higher self-efficacy in women who had previously given birth.

First stage of labour CBSEI subscales were associated with fear [W-DEQ against outcome expectancy and self-efficacy (rho = −.337, p <0.001; rho = −.504, p <0.001)] and depression symptoms [EPDS against outcome expectancy and self-efficacy (rho = −.147, p <0.001; rho = −.243, p <0.001)]. Women with low childbirth efficacy recorded higher fear of birth scores and higher probable depression scores. The correlation was high for fear (W-DEQ) (−.49), and less so for EPDS (−.26).

We found no relationship for age, education, or of having a history of miscarriage against the subscales of outcome expectancy or self-efficacy for first stage of labour in nulliparous or multiparous women. However multiparous women who perceived their partner as not supportive of the pregnancy had significantly lower scores on both outcome expectancy and self-efficacy subscales respectively (m = 101.3, SD = 33.1, t (763) = −2.11, p = .04; m = 96.7, SD = 33.6, t (747) = −2.08, p = .04). However, not having partner support made no difference to self-efficacy levels of nulliparous women (outcome expectancy m = 106.3, SD = 30.6, t (592) = −1.6, p = .11; self-efficacy m = 94.5, SD = 34.2, t (583) = −.76, p = .45) (refer Table 3). Statistically significant associations with lower childbirth efficacy were found for both parity groups against the variables of perceiving less childbirth knowledge compared to peers, preferring a caesarean section for this birth, reporting moderate to extreme pain in second trimester, and having higher EPDS and W-DEQ scores (refer Table 3).

**Table 3:** **Univariate relationships between CBSEI scores and independent variables**

	CBSEI self efficacy				CBSEI outcome			
*Independent variable*	*Nullips = 609*		*Multips = 801*		*Nullips = 609*		*Multips = 801*	
	*t*	*p*	*t*	*p*	*t*	*p*	*t*	*p*
Education level	.70	.49	.30	.76	1.47	.14	.44	.66
≤Year 12 / >Year 12								
Previous miscarriage	.96	.34	.84	.40	1.68	.09	.44	.66
Yes/No								
Level of birth knowledge	3.59	<.001	3.29	.001	2.13	.03	2.46	.01
< peers/								
≥ peers								
Supportive partner	.76	.45	2.08	.04	1.58	.11	2.11	.04
Not at all or indifferent/fairly or very								
Birth preference Vaginal/Caesarean	2.28	.02	2.33	.02	2.53	.01	2.74	.006
Pain in pregnancy none or mild/moderate or severe	2.95	.003	2.08	.04	2.54	.01	1.50	.14
	***r***	***p***	***r***	***p***	***r***	***p***	***r***	***p***
Age	.015	.71	.00	1.0	.05	.25	.03	.37
EPDS	-.26	<.001	-.23	<.001	-.14	.001	-.15	<.001
WDEQ	-.49	<.001	-.49	<.001	-.37	<.001	-.36	<.001

t = t-distribution, p = p-value, r = Pearson correlation co-efficient.

Independent variables were then analysed for correlations against the CBSEI subscales. The same variables that had shown a relationship to first stage CBSEI subscales (Table 3) were also found to be correlated (Tables 4 and 5). However apart from fear (W-DEQ) and depression (EPDS) scores being highly statistically correlated, all other variables had either very small or no correlation. Therefore with most variables showing only small effects, a regression analysis to determine predictors of childbirth efficacy was not conducted.

**Table 4:** **Correlations for independent variables against subscale for outcome expectancy by parity**

	Outcome expectancy 1^st^stage					
Independent variable	Nulliparous n = 609			Multiparous n = 801		
	n (%)	r	p-value	n (%)	r	p-value
Age	602 (99)	.05	.25	773 (97)	.03	.37
Education	599 (98)	.06	.14	773 (97)	.02	.66
Childbirth knowledge	595 (98)	.09*	.03	773 (97)	.09*	.01
History miscarriage	602 (99)	.07	.09	774 (97)	.02	.66
Supportive partner	594 (98)	.07	.11	765 (96)	.10**	.01
Preferred birth	594 (98)	.10*	.01	760 (95)	.10**	.01
Pain 2^nd^ trimester	601 (99)	.10*	.01	772 (96)	.05	.14
WDEQ	597 (98)	.37**	<.001	764 (95)	.36**	<.001
EPDS	602 (99)	.14**	.001	774 (97)	.15**	<.001

**p < .001 (2 tailed).

**Table 5:** **Correlations for independent variables against subscale for self efficacy by parity**

	CBSEI self efficacy 1^st^stage					
Independent variable	Nulliparous n = 609			Multiparous n = 801		
	n (%)	r	p-value	n (%)	r	p-value
Age	593 (97)	.02	.71	757 (95)	.00	1.0
Education	590 (97)	.03	.49	757 (95)	.01	.76
Childbirth knowledge	585 (96)	.15**	<.001	757 (95)	.12**	.001
History miscarriage	593 (97)	.04	.34	758 (95)	.03	.40
Supportive partner	585 (96)	.03	.45	749 (94)	.08*	.04
Preferred birth	586 (96)	.09*	.02	744 (93)	.09*	.02
Pain 2^nd^ trimester	592 (97)	.12**	.003	757 (95)	.08*	.04
WDEQ	589 (97)	.49**	<.001	749 (94)	.49**	<.001
EPDS	593 (97)	.26**	<.001	758 (95)	.23**	<.001

**p < .001 (2 tailed).

## Discussion

We analysed CBSEI scores for first stage of labour sub-scales (15 + 15 items) due to the high reliability and validity between scales in both stages of labour. Other recent studies have either used first stage subscales [5,15,21], second stage subscales [35], or the full 62 item scale [6,36]. In our cohort we found the mean total score for CBSEI subscales to be lower than those reported by Lowe [3] when first testing the CBSEI measure and slightly higher than scores found when tested in a comparable Australian population nearly two decades ago [12]. This is most likely due to the significantly higher number of nulliparous women in Lowe’s study and the higher CBSEI scores found in multiparous women [3,12].

According to self-efficacy theory, previous experience should have the largest impact on self-efficacy, followed by vicarious experience, verbal persuasion/support, and lastly physiological responses such as fear and anxiety [2]. The results of our study support self-efficacy theory as proposed by Bandura [2] and Lowe [3] by demonstrating interactions between childbirth self-efficacy and parity, partner support, knowledge, and fear.

### Socio-demographic variables

Similar to previous studies we found no relationship between demographic variables and childbirth self-efficacy [15,16]. Women’s childbirth self-efficacy may align more to women’s levels of general self-confidence, their existing coping styles and stability of support or role models. Intrinsic personal factors may be more telling than women’s demographic characteristics given the previous finding that prenatal anxiety was strongly associated with lower childbirth self-efficacy [5].

Few previous studies have investigated the role of social support on self-efficacy. The current study found multiparous women reported lower childbirth self-efficacy levels where partner support was lacking compared to first time mothers. Women who have given birth previously may draw on this experience and perhaps recall the value of having a trusted support person in labour to rely on, especially within potentially dehumanising and medicalized birth environments [37]. Fleming and colleagues [37] reported all women feared being left alone in labour, but where multiparous women felt unsupported, their self-efficacy diminished and anxiety increased. Conversely, women having a first baby are likely to enter the maternity environment trusting they will receive the care and support they need from maternity staff, and therefore their reliance on a supportive partner for childbirth success may not have necessarily resonated.

The effect of childbirth knowledge on self-efficacy has also not been investigated to any great degree. Our findings confirm what Drummond and Rickwood [10] reported 20 years previously: that childbirth self-efficacy is higher in women who perceive themselves as more knowledgeable and have previously experienced childbirth. This finding supports the assumption that previous birth knowledge is transferable and explains why nulliparous women may report lower CBSEI scores. Childbirth knowledge is a modifiable variable and worthy of further investigation to enhance women’s confidence for labour and birth.

### Obstetric factors: preference for caesarean section

Consistent with Dilks and Beal [11], we found that levels of self-efficacy for birth were significantly associated with women’s preference for how they would give birth. In our study, nulliparous and multiparous women who had low outcome expectancy (low belief that a behaviour would be helpful) and low self-efficacy scores (lacked confidence to execute the behaviour) for labour and birth were more likely to express a preference for CS. However, these results are in contrast to Salomonsson and colleagues [15] who found that a desire for a CS was related to high childbirth fear and not to low childbirth self-efficacy. Most recently a study in the USA [18] tested a decision self-efficacy measure and found preferences for CS to be related to knowledge. While direct comparison to CBSEI scores cannot be made the common link of birth choice and self-efficacy appears consistent.

### Pain and discomfort in pregnancy

Previous studies have reported a relationship to higher perceived pain levels in labour and low childbirth self-efficacy [3,6]. We are not, however, aware of any previous study linking pain during mid-trimester of pregnancy to childbirth self-efficacy although we found a relationship between second trimester pain and childbirth fear [38]. It is difficult to know if fear comes first or low confidence pre-exists and contributes to this relationship of feeling higher intensity pain during pregnancy compared to other women. This physical manifestation may be a plea for help to cope with labour and birth, or result from feelings of anticipated low coping ability exacerbating normal discomforts of pregnancy. This is an identifiable feature that care providers could use to differentiate from physical causes and explore other reasons for unusual levels of pregnancy discomfort. Supportive strategies to assist women might include strategies that build personal resilience. Support could also be offered through a trusted continuity carer whom the woman knows well and guides them during labour. Care providers could also build women’s knowledge around normality of birth and help to build positive expectations and confidence.

### Psychological factors: depression

There is little doubt that depression, fear, and self-efficacy are related [8]. Indeed in the current study population we previously reported depressive symptoms to be predictive of high childbirth fear [38]. In this current analysis we also found childbirth fear, childbirth self-efficacy and depressive symptoms to be related. Given the adverse consequences of poor perinatal mental health for women and their babies, improving self-efficacy may moderate the development of depression. Depression has been linked with low breastfeeding self-efficacy [39], low parenting self-efficacy [40] and low general self-efficacy [41]. The pervasive negative effect of depression on women’s self-efficacy for childbirth and other motherhood roles, gives support for midwifery continuity of care models where psychosocial issues can be routinely assessed, interventions used to foster positive change, and where indicated, early referral for additional support instituted.

### Childbirth fear

Low CBSEI scores in highly fearful women have been reported as a risk factor for PTSD [24,42]. Childbirth related trauma is an important primary health concern to address given the far reaching and long-term consequences for the family and community. We found childbirth fear scores to be highly correlated to low levels of first stage labour outcome expectancy and self-efficacy. These results are similar to another study looking specifically at severely fearful women that found W-DEQ scores correlated to low level second stage outcome expectancy and self-efficacy subscales [8]. However, these fearful women also reported high outcome expectancy scores, indicating that improving birth knowledge or securing support before and during childbirth modified women’s childbirth self-efficacy [8]. Similarly, addressing childbirth fear through midwifery psycho-education has been found to significantly improve women’s birth confidence [43].

### Implications for practice

Women with low self-efficacy for birth would benefit from increased support in building capacity and developing childbirth coping strategies. Recent research has demonstrated positive outcomes from potential interventions that may serve to increase a woman’s confidence in birth, such as mindfulness training [10], prenatal yoga [36] and increasing women’s knowledge of birth through education [44]. An Australian study reported attendance at a specialised ‘next birth after caesarean’ clinic contributed to an increase in women’s self-efficacy between the booking visit and 36 weeks of pregnancy [19]. The clinic program included time with a midwife to explore feelings and discuss beliefs, provide evidence-based information, and offer continuity of antenatal care.

Similarly, the provision of psycho-education by midwives to fearful women improved their childbirth self-efficacy [43]. Maternity care providers might assist women by working with them to identify and address their worries, individualising care, identifying knowledge and support deficits, and strengthening resilience through information and resources, thus ultimately improving women’s preparation and experience of birth. Providing postnatal women opportunities to discuss and have their birth story heard have also been successful in minimising birth trauma symptoms and foster positive anticipation of a future birth [45] which may impact on self-efficacy.

In addition midwives, doctors and other health care professionals need to reflect on their own beliefs and understanding of labour and birth given the known impact of vicarious experience on women’s childbirth self-efficacy [2,4]. The dominant medical discourses that construct birth as only normal in hindsight do little to address women’s fears and help them prepare in a positive way for the experience of labour and birth [46]. Failing to address women’s sense of childbirth self-efficacy may serve to set them up for a distressing and dissatisfying birth experience.

### Limitations

Women were recruited to the study from metropolitan publicly funded antenatal hospital clinics. This may limit generalizability of the study given 1) women living in rural and remote communities were not included and 2) 30% of Australian women receive private obstetric care. Additionally, social support and knowledge scores were measured using single items. Using a reliable scale for these variables may have shown different outcomes. The study was also restricted to women with a good command of the English language, therefore it is not known if differences would be found for women with language or literacy barriers.

## Conclusions

For all women regardless of parity, significant relationships were found between childbirth self-efficacy, childbirth fear, scoring high for depressive symptoms, low childbirth knowledge, and experiencing a high level of discomfort in pregnancy. For multiparous women, perceiving low partner support for the pregnancy was also important. These key findings underlie the importance of further explorations in this area to determine whether self-efficacy can be used to accurately predict who may benefit from additional education and support during pregnancy to enhance and prepare women for birth and their postnatal maternal experience. Due to the high correlation between childbirth self-efficacy and fear found in this and other studies, and relationships between low childbirth self-efficacy and poor postnatal emotional health reported in the literature, it may be that interventions found to be successful in reducing women’s childbirth fear and birth trauma symptoms may be transferrable to women identified with low childbirth self-efficacy. Increasing levels of childbirth self-efficacy may assist women to approach motherhood more positively, improve their general wellbeing, impact on reducing unnecessary birth interventions, and improve postnatal mental health.

## Abbreviations

BELIEF: Birth emotions looking to improve expectant fear
CBSE: Childbirth self-efficacy
CBSEI: Childbirth self-efficacy inventory
CS: Caesarean section
EPDS: Edinburgh postnatal depression scale
NBAC: Next birth after caesarean
ERCS: Elective repeat caesarean section
PTSD: Post traumatic stress disorder
W-DEQ: Wijma delivery expectancy/experience questionnaire
